# THE EXTREME FACE OF SOCIAL ISOLATION: A COHORT STUDY OF UNBEFRIENDED INDIVIDUALS IN LONG-TERM CARE

**DOI:** 10.1093/geroni/igz038.1957

**Published:** 2019-11-08

**Authors:** Stephanie Chamberlain, Wendy Duggleby, Pamela B Teaster, Janet Fast, Carole Estabrooks

**Affiliations:** 1 University of Alberta, Edmonton, Alberta, Canada; 2 Virginia Tech, Blacksburg, Virginia, United States

## Abstract

Even though social isolation is a significant predictor of poor health and mortality in older adults, very little is known about social isolation in long-term care (LTC) settings. The aim of this study was to describe the prevalence, demographic characteristics, health outcomes, and disease diagnoses of residents without family contact in Alberta LTC homes. Using data collected between April 2008 and March 2018, we conducted a retrospective cohort study using the Resident Assessment Instrument, Minimum Data Set, (RAI-MDS 2.0) data from 34 LTC facilities in Alberta. We identified individuals who had no contact with family or friends. Using descriptive statistics and binary logistic regression, we compared the characteristics, disease diagnoses, and functional status of individuals who had no contact with family and individuals who did have contact with family. We identified a cohort of 25,330 individuals, of whom 945 had no contact with family or friends. Different from residents who had family, the cohort with no contact was younger (81.47 years, SD=11.79), and had a longer length of stay (2.71 years, SD=3.63). For residents who had contact with family, residents with no contact had a greater number of mental health diagnoses, including depression (OR: 1.21, [95% CI: 1.06-1.39]), bipolar disorder (OR: 1.80, [95% CI: 1.22-2.68]), and schizophrenia (OR: 3.9, [95% CI: 2.96-5.14]). Interpretation: Residents without family contact had a number of unique care concerns, including mental health issues and poor health outcomes. These findings have implications for the training of staff and LTC services available to these vulnerable residents.

